# Parkinson’s Disease: Assay of Phosphorylated α-Synuclein in Skin Biopsy for Early Diagnosis and Association with Melanoma

**DOI:** 10.3390/brainsci6020017

**Published:** 2016-05-26

**Authors:** Andrei Surguchov

**Affiliations:** Department of Neurology, Kansas University Medical Center, Kansas City, 3901, Rainbow Boulevard, Kansas City, KS 66160, USA; asurguchov@kumc.edu; Tel.: +1-913-689-0771

**Keywords:** Parkinson’s disease, neurodegeneration, melanoma, synuclein, phosphorylation, diagnosis

## Abstract

Parkinson’s disease (PD) is a degenerative disorder of the central nervous system, in which a small naturally unfolded protein α-synuclein plays an essential role. α-Synuclein belongs to a synuclein family comprising three members: α, β, and γ-synucleins associated with neurodegenerative and neoplastic diseases and involved in development. Several studies revealed that α-synuclein is present not only in the brain, but also in the skin and other peripheral tissues. This finding open a new approach to PD diagnosis based on the assay of α-synuclein from a biological sample of a living patient. Furthermore, PD is associated with an increased risk of skin melanoma. An important posttranslational modification of α-synuclein is phosphorylation at serine-129, which may convert the protein into pathological species both in PD and melanoma. Thus, analysis of phosphorylated α-synuclein might be an important diagnostic test for both diseases providing additional information about the mechanism of pathology.

Parkinson’s disease (PD) is a common severe neurodegenerative disorder that affects a significant proportion of the adult population. The risk of developing PD is age dependent, affecting 1%–2% of the population over 65 years [1,2]. PD leads to a decline in motor, mental, and functional skills and is associated with significantly higher mortality rates. PD has been defined by a loss of the neurotransmitter dopamine due to premature death of dopaminergic neurons in the brain and the presence of α-synuclein containing Lewy bodies (LBs) and Lewy neurites (LNs) [3]. The underlying pathophysiology includes progressive gradual destruction of several brain regions, including the brain stem, the forebrain, the extrapyramidal system, and later the cortical areas [4].

PD has a long preclinical stage during which important information about the disease usually remains overlooked. Late diagnosis and misdiagnosis of PD are common, emphasizing the requirement for disease-specific and early stage biomarkers. Both early diagnosis of PD and adequate tracking of disease progression could improve outcomes for patients, from the point of view of existing and upcoming disease modifying treatments. Many attempts have been made to find biomarkers at early stages of PD, using analysis of blood, serum, plasma, CSF, skin, saline, urine, *etc.* (reviewed in [5]). However, none of the tested techniques provided a reliable and convenient method and its diagnosis is primarily based on motor-related clinical criteria. There is an urgent need to identify biomarker(s) for an early diagnosis of PD, preferably during the pre-motor phase.

One of the key proteins in PD pathogenesis is α-synuclein (reviewed in [6]), being the major constituent of the PD hallmarks, LBs and LNs. These insoluble protein aggregates do not themselves have a prominent neurotoxic effect, whereas intermediate oligomeric forms of α-synuclein appear harmful. α-Synuclein pathology is closely associated to the degenerative process [7]. Although the function of α-synuclein is not completely understood, it is primarily localized to the presynaptic terminals of mature neurons, where it fulfills roles in synaptic function and plasticity. The results of several studies point to its role in maintaining a supply of synaptic vesicles in presynaptic terminals by clustering synaptic vesicles. Importantly, it is also involved in the release of dopamine, a neurotransmitter that is critical for controlling the start and stop of voluntary and involuntary movements. By altering the correct action of key molecules involved in the control of neurotransmitter release and re-cycling, as well as synaptic and structural plasticity, α-synuclein deposition may crucially impair axonal trafficking, accompanied by a sequence of harmful events, ultimately leading to neuronal degeneration and death. The pathological alterations in functional and structural synaptic plasticity impairs learning mechanisms, motor performance and memory. It is however not clear how the dopamine loss in these patients leads to a disruption in motor complex plasticity.

The use of α-synuclein as a PD marker is relatively easy, because it is present not only in the brain, but also in peripheral organ and tissues (Figure 1).

Currently, the study of α-synuclein phosphorylation mechanism related to the PD pathology has become a research hotspot, because after phosphorylation by G-protein-coupled kinase [9] or other kinases this protein alters its conformation and becomes toxic. α-Synuclein has at least three experimentally proven phosphorylation sites, however, phosphorylation of serine residue at position 129 (Ser129) alters physiological properties of the protein most drastically. Importantly, 90% of α-synuclein deposition in LBs is phosphorylated at Ser129, whereas in normal brains, only 4% or less of α-synuclein is phosphorylated at this residue [10,11]. Since α-synuclein phosphorylation converts it into pathological species, this mechanism became a subject of intensive investigation. According to recent data, LBs may also contain another member of the synuclein family, γ-synuclein oxidized at methionine-38 (Met-38) [12,13,14].

α-Synuclein is a naturally unfolded protein easily changing its conformation and only specific conformers possess toxic properties. Thus, generation of conformation-specific α-synuclein antibodies and/or antibodies distinguishing phosphorylated/nonphosphorylated α-synuclein is an important step for the improved specificity of PD diagnosis [15]. Hopefully, these antibodies may also be used for immunotherapy of PD and related pathologies.

PD diagnosis based on α-synuclein analysis may be simplified, because this protein is present not only in the brain, but also in peripheral tissues (Figure 1). Several attempts to use α-synuclein analysis for early diagnosis of PD, including its testing in serum, plasma, cerebrospinal fluid (CSF), CNS-derived extracellular vesicles (EVs) in plasma, *etc.*, have been made [16,17,18,19,20] (reviewed in [5]).

Initially testing of α-synuclein in CSF as early PD diagnostic tool gave promising results [20,21], but relatively invasive method for collecting CSF is not appropriate in most clinical settings. An important step forward for better understanding of PD pathogenesis and development of early biomarkers of this disease was finding of α-synuclein histopathology in the peripheral tissues, including peripheral autonomic nervous system [8,22,23,24,25]. In living PD patients, α-synuclein pathology has been described in minor salivary glands, submandibular gland, stomach, colon, olfactory epithelium, skin and other tissues and body fluids (Figure 1) [22]. The use of primary skin fibroblast cultures for PD diagnostic test based on the measurements of stress–induced gene expression changes [22] gave hope that a transcriptome approach to a readily accessible peripheral tissue might detect useful biomarkers for PD patient diagnostics and offer a clue to key steps in pathology process. In other studies many differences have been found in skin fibroblasts taken from PD patients compared to control individuals in addition to increased α-synuclein expression [22], including altered protein expression pattern [26], bioenergetic and proteolytic defects [27], mitochondrial dysfunction [28], the decreased activity of complex V and other changes in oxidative stress [29], enhanced vulnerability of PARK6 patient skin fibroblasts to apoptosis [30] and respiratory chain defects [31].

Recently, an important step forward in PD early diagnosis is described by Donadio and coauthors [32]. These researchers have found that the presence of inclusions of phosphorylated α-synuclein in the skin sympathetic nerve fibers may be used as a sensitive *in vivo* biomarker for degenerative peripheral autonomic neuropathy. In a later study, the same research team applied similar method for analysis of native α-synuclein (n-syn) and misfolded phosphorylated (p-syn) α-synuclein in idiopathic Parkinson disease (IPD) attempting to define the importance of n-syn and p-syn for PD diagnosis [33]. The authors also compared n-syn and p-syn in patients with IPD and pure autonomic failure (PAF) and found length-dependent somatic and autonomic small fiber loss in IPD patients, more severely expressed in patients with higher p-syn load. Furthermore, p-syn was not detected in skin sample of control individuals, but was found in all IPD patients. Thus, p-syn may be used as an *in vivo* marker of IPD, and its analysis in skin shows that p-syn inclusions are different in PAF and IPD, pointing to dissimilar underlying pathogenic mechanisms [33].

Therefore, recent studies have shown that methods based on the use of n-syn/p-syn ratio analysis in skin biopsy to diagnose PD and other synucleinopathies *in vivo*, are promising because they are based on a direct, straightforward, low-priced, and minimally invasive technique with minor discomfort for the patient.

Importantly, patients with PD have an increased risk of developing melanoma, which is the major cause of skin cancer death worldwide [34]. It has been found in several epidemiologic studies that the occurrence of PD in melanoma patients or the occurrence of melanoma in PD patients is significantly higher than expected (reviewed in [35]). A high level of α-synuclein expression has been identified in human malignant melanoma tumors and cell lines [34]. Although α-synuclein is primarily a neuronal protein, its elevated expression is found in various tumors, including ovarian, colorectal and melanoma tumors suggesting that neurodegeneration may share common mechanisms with oncogenesis [35]. The exact reason of the puzzling association between PD and melanoma, including a shared risk and overlapping disease mechanisms remains undefined [36,37]. Intriguingly, the frequency of most other cancers is lower in PD patients compared to the general population [38].

Several putative players may be involved in this association, including tyrosine, tyrosinase, tyrosine hydroxylase, DJ-1 and melanocortin 1 receptor [36,37,38,39]. An interesting hypothesis is that phosphorylation of Ser129 in α-synuclein plays a role in transition of this protein to pathological species not only in PD, but also in melanoma [40]. According to Lee and coauthors, the Ser129 phosphorylated form, but not the Ser129-unphosphorylated form of α-synuclein localizes to dot-like structures at the cell surface and the extracellular space of melanoma cells [40]. Phosphorylation of Ser129 regulates the release of α-synuclein through the vesicular trafficking and leads to the cell surface translocation of α-synuclein along the microtubule network and subsequent vesicular release both in melanoma and neuronal cells [39,40]. Importantly, α-synuclein in specific conformation released from one cell may be taken by another, thus spreading the pathology by prion-like mechanism. Thus, phosphorylation of α-synuclein is a mechanism controlling its transition from native to pathological conformations, inducing neurotoxicity and inclusion formation. Phosphorylation may be a trigger changing other α-synuclein properties important for its role in pathology. For instance, phosphorylation increases metal ion binding affinity, for example, for Cu (II) and Fe (II); it also affects long-range interactions of α-synuclein C- and N-termini [41]. Phosphorylation as a mechanism changing α-synuclein properties is evolutionary conserved and operates in Drosophila model of PD [42].

An important ongoing international multicenter prospective study The Parkinson’s Progression Markers Initiative (PPMI) [43] is aimed to validate biomarkers in drug-naïve PD patients and matched healthy controls. Quantification of CSF α-synuclein, amyloid-beta1-42, total tau (t-tau), and tau phosphorylated at Thr181 (p-tau) and subsequent correlation of these results with measures of the clinical features of these subjects brought interesting results. For example, CSF α-synuclein, t-tau and p-tau levels, were significantly lower in PD compared with healthy controls. However, the diagnostic value of the individual CSF biomarkers for PD diagnosis was limited due to large overlap [44].

The sensitivity and specificity of p-syn analysis in skin and other peripheral tissues in some studies is not high enough to use it as a routine method of PD diagnostic [45,46], which can be explained, at least partially, by diversity of methods used, including tissue processing, protocols details , variety in evaluation of the findings, *etc.* However, it is usually better with p-syn than with α-synuclein. For example, Wang and coauthors [47] concluded that specificity of p-syn was satisfactory (absent staining in controls), whereas α-synuclein immunohistochemistry showed immunoreactive signals both in PD patients and in controls, however, with increased α-synuclein deposition in PD patients [47]. Some modifications of the protocol may improve the sensitivity and specificity of p-syn analysis. For example, Beach and colleagues [48] proposed to include in the protocol the use of proteinase K pretreatment to increase the specificity. Furthermore, optimization of the time of such treatment may be necessary, because proteinase K potency may vary from batch to batch [48]. The development of a new sandwich-type ELISA for measuring total-, oligomeric- and phosphorylated-Ser129 [18] may be an important step forward for improvement of PD early diagnosis.

Thus, Ser129 phosphorylated α-synuclein may be a more reliable marker compared to unphosphorylated protein, and its analysis demonstrates higher specificity and better prognostic value. Collecting of skin samples is inexpensive and not invasive method. Therefore, the analysis of phosphorylated α-synuclein can be used as an important diagnostic test for both diseases and provide additional information about the mechanism of pathology.

## Figures and Tables

**Figure 1:**
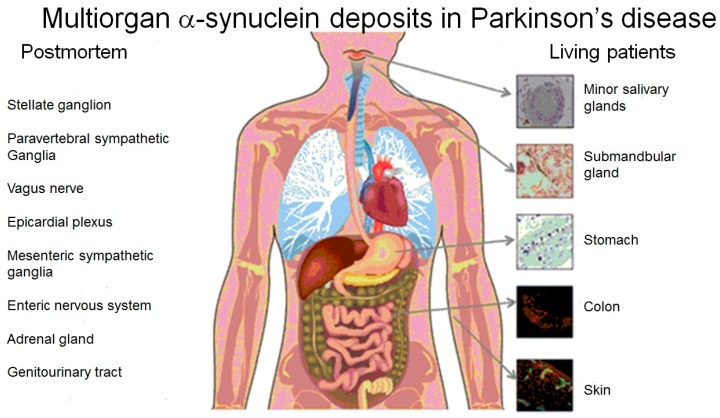
Peripheral tissues in which phosphorylated α-synuclein deposits have been reported to occur in Parkinson’s disease (taken with permission from [8]).
